# Phylogeography and evolutionary dynamics of Nairobi sheep disease virus

**DOI:** 10.1093/ve/veag061

**Published:** 2026-09-21

**Authors:** Han-Zheng Mu, Si-Qian Wu, Mei-Qi Zhang, Gui-Yan Shi, Jia-Yi Wang, Ming Chen, Yun-Dong Shi, Hong Su, Xiao-Ai Zhang, Wei Liu

**Affiliations:** State Key Laboratory of Pathogen and Biosecurity, Academy of Military Medical Science, 20 Dong-Da Street, Fengtai District, Beijing 100071, China; State Key Laboratory of Pathogen and Biosecurity, Academy of Military Medical Science, 20 Dong-Da Street, Fengtai District, Beijing 100071, China; State Key Laboratory of Pathogen and Biosecurity, Academy of Military Medical Science, 20 Dong-Da Street, Fengtai District, Beijing 100071, China; State Key Laboratory of Pathogen and Biosecurity, Academy of Military Medical Science, 20 Dong-Da Street, Fengtai District, Beijing 100071, China; State Key Laboratory of Pathogen and Biosecurity, Academy of Military Medical Science, 20 Dong-Da Street, Fengtai District, Beijing 100071, China; State Key Laboratory of Pathogen and Biosecurity, Academy of Military Medical Science, 20 Dong-Da Street, Fengtai District, Beijing 100071, China; State Key Laboratory of Pathogen and Biosecurity, Academy of Military Medical Science, 20 Dong-Da Street, Fengtai District, Beijing 100071, China; Department of Epidemiology and Health Statistics, School of Public Health, Anhui Medical University, 81 Mei-Shan Road, Shushan District, Hefei 230032, China; Department of Epidemiology and Health Statistics, School of Public Health, Anhui Medical University, 81 Mei-Shan Road, Shushan District, Hefei 230032, China; State Key Laboratory of Pathogen and Biosecurity, Academy of Military Medical Science, 20 Dong-Da Street, Fengtai District, Beijing 100071, China; State Key Laboratory of Pathogen and Biosecurity, Academy of Military Medical Science, 20 Dong-Da Street, Fengtai District, Beijing 100071, China; Department of Epidemiology and Health Statistics, School of Public Health, Anhui Medical University, 81 Mei-Shan Road, Shushan District, Hefei 230032, China; The First Affiliated Hospital of Anhui Medical University, 218 Ji-Xi Road, Shushan District, Hefei 230032, China

**Keywords:** Nairobi sheep disease virus, molecular evolution, reassortment and recombination, selection pressure, phylogeography

## Abstract

Nairobi sheep disease virus (NSDV) is a tick-borne *orthonairovirus* causing severe hemorrhagic gastroenteritis in small ruminants, with case fatality rate up to 90% among naive populations. Despite its veterinary and zoonotic importance, the evolution and spatiotemporal dynamics of NSDV remain poorly understood. We analysed S, M, and L segment sequences collected from the 1950s to 2024 in China, India, and Kenya, and identified three geographically structured lineages (Lineage A from India, Lineage B from China, and Lineage C from Kenya). No host-specific clustering was observed, indicating geographic isolation, rather than host adaptation, is the primary driver of diversification. Recombination events exhibited strong geographic structuring, with Jilin province as a major hotspot (likely reflecting higher sampling density in the region). Occasional intercontinental gene flow from Africa into China contributed parental lineages, and recombinant strains were detected in ticks, with parental contributions from sheep and saiga antelope hosts. Selection analyses revealed strong purifying selection on L segment (dN/dS = 0.365), but relaxed or positive selection on M glycoprotein (dN/dS = 3.020) and S segment (dN/dS = 5.360). Substitution rates ranged from 1.56 × 10^−4^ (L) to 6.81 × 10^−4^ (S) substitutions/site/year. Time-to-most-recent-common-ancestor (tMRCA) of all NSDV strains was estimated at ~ 1732 ce (95% highest posterior density [HPD]: 1624–1833), representing the divergence between the Chinese lineage and the common ancestor of the Indian and Kenyan lineages. The Indian and Kenyan lineages subsequently diverged from their common ancestor ~ 1763 ce (95% HPD: 1677–1845), followed by the Indian lineage (~1883 ce) and the Chinese lineage (~1934 ce). The ancestral location at the root node is highly uncertain (maximum *a posteriori*, MAP = India_Karnataka), and sparse sampling from intermediate regions limits robust phylogeographic inference. Among the well-supported intra-continental routes, migration from India (Maharashtra/Karnataka) to eastern China (Liaoning, Shandong) received moderate support (BF = 6.40–22.57). A route from Liaoning to Kenya was also detected (BF = 11.84), but this inference should be interpreted with caution given the limited Kenyan sampling and potential unsampled intermediate populations.

## Introduction

Nairobi sheep disease virus (NSDV) is a tick-borne *orthonairovirus* (family *Nairoviridae*, order *Bunyavirales*) and the causative agent of Nairobi sheep disease (NSD) (Kuhn et al. 2024). NSD is characterized by severe hemorrhagic gastroenteritis and lymphoid depletion, with case fatality rates ranging from 25% to over 90% in susceptible naive sheep and goat population (Yao et al. 2025). Historically confined to East Africa and South Asia, NSDV was first recognized as a significant pathogen in Kenya in 1910 (Montgomery 1917), and subsequently spread widely across the African continent (Weinbren et al. 1958, Edelsten 1975, Davies et al. 1978). Ganjam virus, considered the Asian variant of NSDV, was first isolated in Asia in 1954 from *Haemaphysalis intermedia* ticks in India (Dandawate and Shah 1969), and was also reported in Sri Lanka in 1996 (Perera et al. 1996). Although NSDV is primarily recognized as a devastating pathogen of small ruminants, evidence of human infection and exposure in high-risk populations has increasingly been reported. The first human infection was recorded in a 12-year-old boy in 1969 (Dandawate et al. 1969) and several laboratory-acquired infections have been documented subsequently (Rao et al. 1981). Notably, NSDV is phylogenetically closely related to the highly pathogenic Crimean-Congo hemorrhagic fever virus (Lasecka and Baron 2013).

Ticks serve as the primary vectors and reservoirs of NSDV. In Africa, *Rhipicephalus appendiculatus* is the main vector, while *H. intermedia* fulfils this role in South Asia (Baron and Holzer 2015, Hartlaub et al. 2021). In recent decades, increasing global animal movement and the spread of tick vectors, predominantly *R. appendiculatus* in Africa and *H. intermedia* in India, have driven the expansion of NSDV’s geographical range (Baron and Holzer 2015, Hartlaub et al. 2021). In China, NSDV RNA was first detected in *Haemaphysalis longicornis* collected from Liaoning, Jilin, and Heilongjiang provinces in 2013 (Gong et al. 2015). Subsequently, the virus has been identified in multiple geographic regions, including Hubei, Henan and Shandong province (Yang et al. 2019, Wang et al. 2024, Yao et al. 2025), and natural infection in Chinese sheep populations has been definitively confirmed (Yao et al. 2025). Genetically, Chinese NSDV strains form a distinct phylogenetic clade separate from African and Indian strains (Gong et al. 2015, Yang et al. 2019, Wang et al. 2024).

Despite its substantial veterinary and potential public health importance, critical gaps remain in our understanding of NSDV evolution and pathogenesis (Zhang et al. 2026). Specifically, systematic studies on genetic reassortment and recombination, key processes shaping viral diversity and adaptive evolution, are scarce, with no comprehensive analyses of reassortment frequencies, genomic hotspots, or their functional consequences. Furthermore, phylogeographic studies tracing the geographic origins, dispersal routes, and spatiotemporal dynamics of NSDV lineages across its global range are lacking, limiting our ability to understand how ecological factors drive viral expansion and lineage replacement.

In the current study, by integrating global NSDV sequences since its discovery, we performed a comprehensive analysis of the virus’s evolutionary dynamics and transmission patterns. We aimed to characterize the phylogenetic structure and geographic lineages of NSDV; to evaluate the roles of reassortment and recombination in generating genetic diversity; to identify selection pressures acting on different genomic segments; and to elucidate its geographic origins and transmission routes.

## Materials and Methods

### Sequence data collection and preparation

We compiled a comprehensive dataset of publicly available NSDV sequences. All complete or near-complete S, M, and L segment sequences were retrieved from GenBank. Sequences lacking precise geographic origin or collection date were excluded (n = 2 L-segment sequences), yielding final datasets of 49 L, 33 M, and 76 S segment sequences. To mitigate sampling bias and reduce sequence redundancy, each segment dataset was independently clustered using CD-HIT-EST with a stringent sequence identity threshold of 99.8% (Fu et al. 2012). The final datasets comprised 49 L segment, 33 M segment, and 76 S segment sequences. Detailed information for each sequence, including host species, sampling location, and collection date, was composed (Supplementary Table 1). Multiple sequence alignments for each segment were generated using MAFFT (v7.520) (Katoh and Standley 2013) and subsequently refined manually in MEGA (v11) (Tamura et al. 2021) to ensure alignment quality and correct reading frames.

### Phylogenetic inference

The best-fitting nucleotide substitution model for each of the three segment alignments was selected using ModelFinder (Kalyaanamoorthy et al. 2017) based on the Bayesian Information Criterion (BIC). Phylogenetic relationships were reconstructed using two complementary approaches. First, maximum likelihood (ML) trees were inferred with IQ-TREE (v2.0.3) (Minh et al. 2020), employing 1 000 ultrafast bootstrap approximations to assess nodal support. Second, Bayesian inference (BI) was performed using MrBayes (v3.2.7) (Ronquist and Huelsenbeck 2003). For each BI analysis, two independent Markov chain Monte Carlo (MCMC) runs were conducted for 200 million generations, sampling trees every 1 000 generations. Convergence was assessed by ensuring effective sample sizes (ESS) exceeded 200 and by examining the potential scale reduction factor; the first 10% of samples were discarded as burn-in. The resulting phylogenetic trees were visualized and annotated in FigTree (v1.4.5).

### Reassortment analysis

To investigate potential genomic reassortment among the three segments (L, M, S) of NSDV, we performed topological incongruence analysis combined with permutation test based statistical validation, following a previously described framework for segmented RNA viruses with minor modifications (Pérez et al. 2024). ML phylogenetic trees of the NSDV L, M, and S segments (18 strains) were constructed using the methods described in the previous section, employing IQ-TREE (v2.0.3) with the best fit GTR + G + I substitution model. Trees were pruned to retain only common strains *via* ‘keep.tip’ (ape v5.7 1) and ladderized for standardized comparison. Cophenetic distance matrices were computed for each segment pair (phangorn v2.11.1), and the maximum cophenetic difference was calculated for each strain to quantify topological divergence. A permutation test (1 000 permutations, fixed seed = 123) was implemented to generate null distributions, with two significance thresholds defined: a strain specific 95% quantile threshold (stringent criterion, *P* < 0.05) and a unified global threshold (0.528, mean of three segment pair global 95% quantiles) for complementary screening. Topological incongruence was visualized *via* custom tanglegram plots, and Robinson Foulds (RF) distances were calculated to quantify overall tree dissimilarity. All analyses were performed in R (v4.3.1) with dplyr (v1.1.4) for data processing.

### Recombination analysis

To identify intra-segment homologous recombination events, we employed a two-step screening and validation approach following established viral evolutionary analysis protocols. Nucleotide sequences of the L, M, and S segments from 18 NSDV isolates were separately aligned using MAFFT (v7.520) (Katoh et al. 2002) with the L-INS-i algorithm and manually curated in AliView (v3.0) (Larsson 2014) to remove poorly aligned regions. Initial recombination screening was performed using Recombination Detection Program 4 (RDP4, v4.101) (Martin et al. 2015) with seven independent methods: RDP, GENECONV, Bootscan, MaxChi, Chimaera, SiScan, and 3Seq. A Bonferroni corrected significance threshold of *P* < 0.05 was used, and only events detected by at least three methods were designated as preliminary candidates. All preliminary candidates were rigorously validated using Simplot (Samson et al. 2022): a sliding window analysis (200 bp window, 20 bp step) was performed to detect significant sequence similarity shifts (≥10%) between the recombinant and its predicted major/minor parents, and phylogenetic trees of regions flanking predicted breakpoints were constructed to confirm topological incongruence. Only events meeting both RDP4 (≥3 methods, *P* < 0.05) and Simplot (similarity shift + topological incongruence) criteria were classified as confirmed recombination events.

### Selection pressure analysis

To investigate the evolutionary constraints acting on the NSDV genome, we performed selection pressure analyses on the coding regions of the L, M, and S segments. Following the exclusion of recombinant sequences, a total of 11 complete genome sequences were aligned at the nucleotide level for each segment. To ensure comprehensive coverage of the complete open reading frames (ORFs), sequences were filtered to retain those with a minimum length of 1400 nucleotides. The ratio of nonsynonymous to synonymous substitution rates (dN/dS, ω) was estimated using the codeml program implemented in the PAML package (Yang 2007). For each genomic segment, we applied: Model 0 (one-ratio model): to estimate the overall mean ω ratio across all codon sites, providing a genome-wide measure of selective constraint. Site-specific models (M1a, M2a, M7, and M8): to identify individual codons evolving under positive selection. These models allow ω to vary among sites and detect signatures of diversifying selection. Likelihood ratio tests (LRTs) were employed to compare nested models (M1a versus M2a; M7 versus M8), with significant *P*-values indicating the presence of sites under positive selection. For codons identified as candidates for positive selection, Bayes Empirical Bayes (BEB) analysis was conducted to calculate posterior probabilities and identify specific sites with high confidence (posterior probability > 0.95). In parallel, nucleotide sequence variability was quantified using Shannon entropy analysis. For each alignment position, entropy (H) was calculated using the formula:


$$H=-\sum \limits_{i=1}^4\mathrm{p}i{\log}_2\left(\mathrm{p}i\right)$$

where pi represents the frequency of each nucleotide (A, C, G, or T) at a given site. Sites with gaps or ambiguous bases were excluded from the calculation to ensure data quality. This approach enabled identification of highly polymorphic genomic regions that may represent targets of host immune pressure or functional adaptation.

### Phylodynamic and phylogeographic analysis

To elucidate the evolutionary history and spatial dynamics of the NSDV, we conducted Bayesian phylogenetic inference using BEAST (v1.10.4) (Suchard et al. 2018). Analyses were performed on concatenated or individual segment datasets (S, M, L) under a GTR + Γ nucleotide substitution model, selected *via* ModelTest-NG (Darriba et al. 2020) based on the Bayesian Information Criterion (BIC). Prior to clock model selection, we rigorously assessed temporal signal using TempEST (v1.5.3). The analysis revealed a strong linear correlation between sampling time and root-to-tip divergence (correlation coefficient r = 0.94, *R*^2^ = 0.89, residual mean square ≈ 7.76 × 10^−4^; Supplementary Fig. 1), confirming clock-like evolutionary behaviour and justifying the use of a strict molecular clock. Temporal signal was calibrated using the sampling year of each sequence (tip dates), and demographic history was co-estimated using a coalescent Bayesian skyline prior. Four independent MCMC chains were run for 100 million generations each, with parameters sampled every 10 000 steps. Convergence and stationarity were rigorously assessed in Tracer (v1.7) (Rambaut et al. 2018), ensuring all parameters achieved ESS greater than 200. After discarding 10% of samples as burn-in, a maximum clade credibility (MCC) tree was summarized using TreeAnnotator (v1.10.4), with node heights scaled to the mean. Mean evolutionary rates (substitutions/site/year) and their 95% highest posterior density (HPD) intervals were extracted for each genomic segment. To reconstruct the spatial dynamics of NSDV, we implemented a discrete phylogeographic model in BEAST, treating sampling locations as a categorical trait. Locations were defined as: China (Beijing, Heilongjiang, Liaoning, Shandong, Hubei, Henan), India (Karnataka, Maharashtra, Orissa), and Kenya. A Bayesian stochastic search variable selection (BSSVS) procedure was applied to identify statistically supported migration routes. Support for each possible transition was quantified *via* Bayes Factor (BF) and posterior probability (PP); routes with BF > 2 and PP > 0.5 were considered well-supported diffusion pathways. The resulting spatial dynamics were visualized using SpreaD3 (v0.9.7.1) (Bielejec et al. 2016) and the R package ggspatial, with line widths proportional to PP and colours scaled to log₁₀(BF).

### Statistical analysis

All statistical tests were two-tailed with a significance level of α = 0.05. Permutation tests and sequence alignments were performed with fixed parameters to ensure reproducibility. All statistical analyses and data visualization were conducted in R (v4.3.1) using the packages ggplot2, ggpubr, ape, and seqinr.

## Results

### Phylogenetic structure and geographic lineages

We reconstructed ML phylogenies for the S, M, and L segments of NSDV, using a curated dataset of high-quality, complete or near-complete sequences (n = 158 across three segments), including recent Chinese isolates, historical Indian and Kenyan isolates. All three segment trees consistently resolved three well-supported, geographically structured genetic lineages (bootstrap support > 70% for all major nodes): Lineage A, Lineage B, and Lineage C (Fig. 1). Lineage A comprises historical Indian isolates (collected between the 1950s and 2000s) and forms a basal monophyletic clade. Lineage B consists exclusively of recent Chinese isolates (collected from 2013 to 2024) and has undergone marked expansion in recent years. Lineage C comprises Kenyan isolates and is evolutionarily closest to Lineage A.

**Figure 1:**
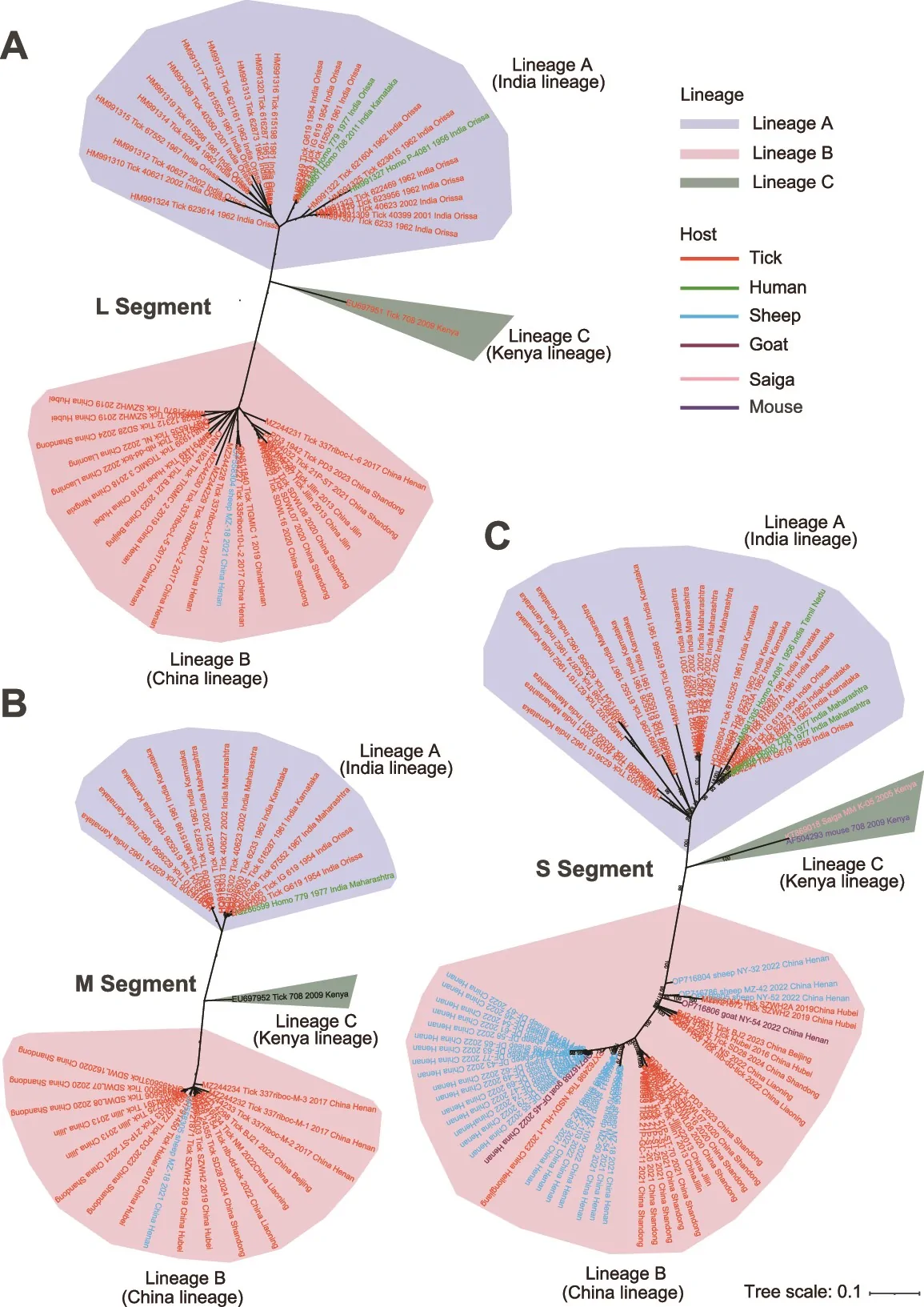
Phylogenetic analysis of the L, M, S segments of NSDV. Maximum-likelihood phylogenetic tree of complete L, M, S segments of NSDV, constructed using the GTR + G + I nucleotide substitution model with 1 000 bootstrap replicates. (A) L segment. (B) M segment. (C) S segment. The tree is rooted at the midpoint, and bootstrap values ≥70% are shown at the nodes. The scale bar represents 0.1 nucleotide substitutions per site. Three distinct phylogenetic lineages are annotated: Lineage A (India lineage, light blue), Lineage B (China lineage, light red), and Lineage C (Kenya lineage, green). Sequence labels include GenBank accession number, host/sample type, isolate name, collection year, and geographic origin. Host-specific colours are indicated in the legend: red = Tick, green = Human, blue = Sheep, purple = Goat, light pink = Saiga, dark blue = Mouse.

No host-specific clustering was observed; sequences derived from ticks, sheep, goats, humans, mice, and saiga antelope were intermingled within each lineage. In Lineage B, tick-derived strains co-clustered with sheep- and goat-derived strains, while human isolates from India fell within Lineage A alongside tick isolates (Supplementary Figs. 2–4). This pattern is consistent with NSDV’s broad arthropod–vertebrate transmission cycle, in which cross-species spillover is frequent and host-specific evolutionary constraints are minimal.

Pairwise genetic distances (Kimura2-parameter model) among the three geographical lineages were markedly higher than intra-lineage distances, supporting obvious lineage-level genetic differentiation of NSDV. For the S segment, inter-lineage genetic distances ranged from 0.11 to 0.23, while intra-lineage distances were only 0.04–0.08. These inter-lineage distances are comparable to those reported among established genotypes of other *Orthonairovirus* species (e.g. CCHFV). The detailed pairwise distance statistics are summarized in Supplementary Table 2.

### Reassortment events in NSDV

To investigate potential inter-segment reassortment, we reconstructed ML phylogenies for each genomic segment (L, M, and S) using the 18 NSDV strains with complete tripartite genome sequences (Fig. 2). Tanglegrams and RF distance calculations revealed substantial topological discordance across the three segment trees: L versus M (RF = 0.72), L versus S (RF = 0.38), and M versus S (RF = 0.71). Such discordance is often considered suggestive of reassortment in segmented RNA viruses; however, it can also arise from homoplasy, lineage-specific rate variation, or stochastic phylogenetic error. To formally test for reassortment, we performed a permutation test using strain-specific 95% quantile thresholds of the maximum cophenetic distance difference. A complementary screening using a uniform global threshold (0.528) identified four potential candidate strains (708, 779, IG619, G619) that exceeded this threshold in the L versus M and/or M versus S comparisons. However, the candidates did not exceed the threshold in the L versus S segment pairs (L versus S values [0.187–0.201] below the L versus S threshold of 0.206), and none met the more stringent strain-specific 95% quantile criteria (all strain-specific *P* > 0.05; Supplementary Table 3). Therefore, no statistically supported inter-segment reassortment was detected among the 18 complete genomes analysed, and the observed topological discordance may be better explained by stochastic lineage sorting or localized selection than *bona fide* reassortment. It should be noted that this conclusion is limited to the 18 complete genomes analysed; the detection of reassortment may be constrained by the small sample size, and the possibility of reassortment in NSDV cannot be excluded with the current data.

**Figure 2:**
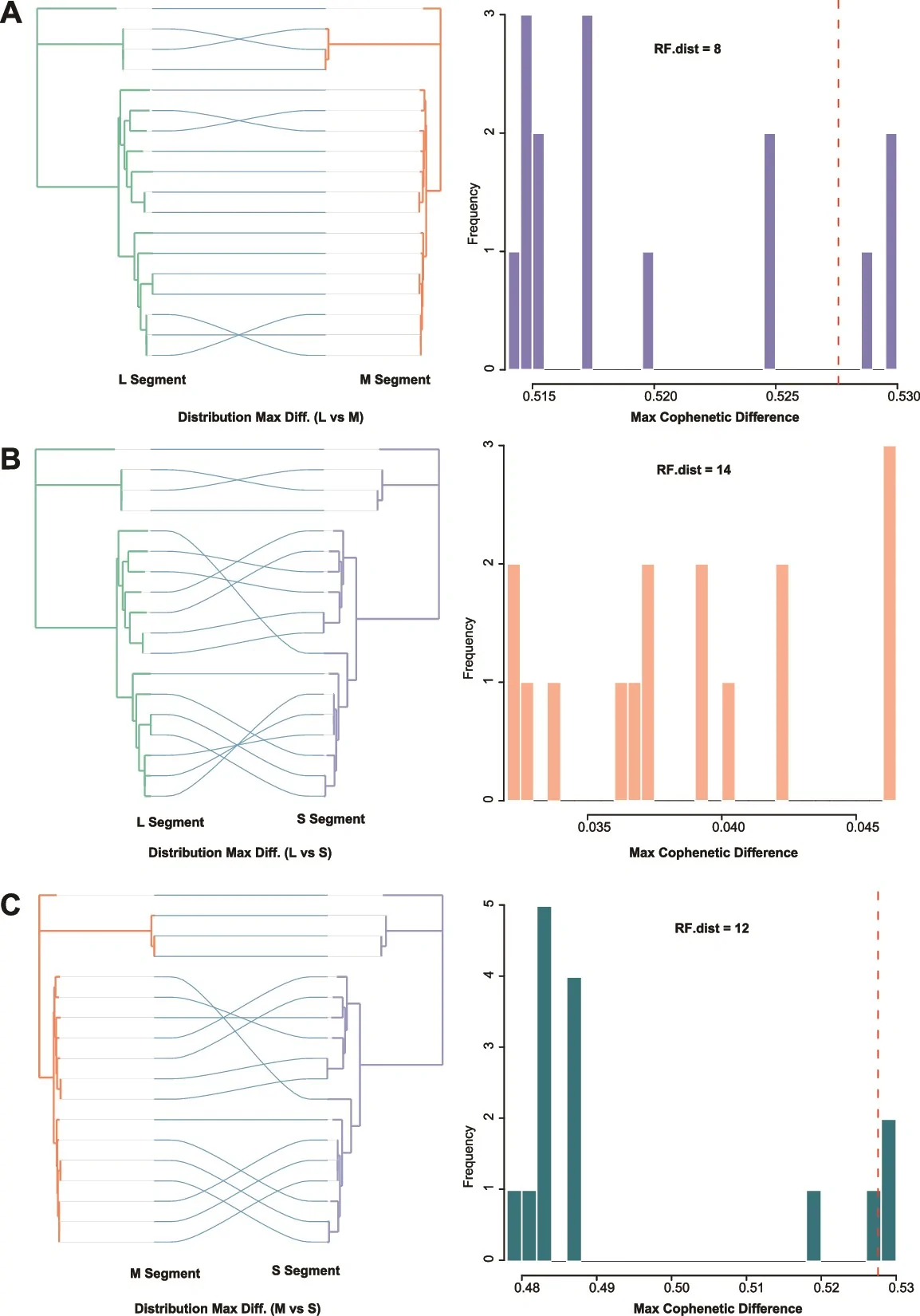
Phylogenetic congruence analysis of NSDV L, M, and S genomic segments. Phylogenetic congruence and topological discordance analysis of NSDV genomic segments. (A) Comparison of L and M segment ML phylogenies, visualized with tanglegrams (left panel) and distribution of maximum cophenetic differences (right panel). The observed RF (Robinson-Foulds) distance was eight, with the red dashed line marking the empirical maximum cophenetic difference threshold. (B) Comparison of L and S segment ML phylogenies, with an observed RF distance of 14. (C) Comparison of M and S segment ML phylogenies, with an observed RF distance of 12. Tanglegrams illustrate topological discordance between segment trees, while histograms show the frequency distribution of maximum cophenetic differences from 1000 random tree permutations, with the red dashed line indicating the observed value for the real dataset. Strain-specific *P*-values > 0.05 indicate no statistically significant reassortment (α = 0.05). All ML trees were constructed using the GTR + G + I nucleotide substitution model with 1 000 ultrafast bootstrap replicates.

### Homologous Recombination

A total of 10 confirmed homologous recombination events were identified across the L, M, and S segments (Fig. 3, Supplementary Table 4). The L segment contained the highest number of events (n = 5), followed by the S (n = 3) and M (n = 2) segments. All recombinant strains were derived from ticks, while their parental sequences were identified from ticks, sheep, or saiga antelope. This pattern is consistent with the circulation of recombinant NSDV across different host species.

**Figure 3:**
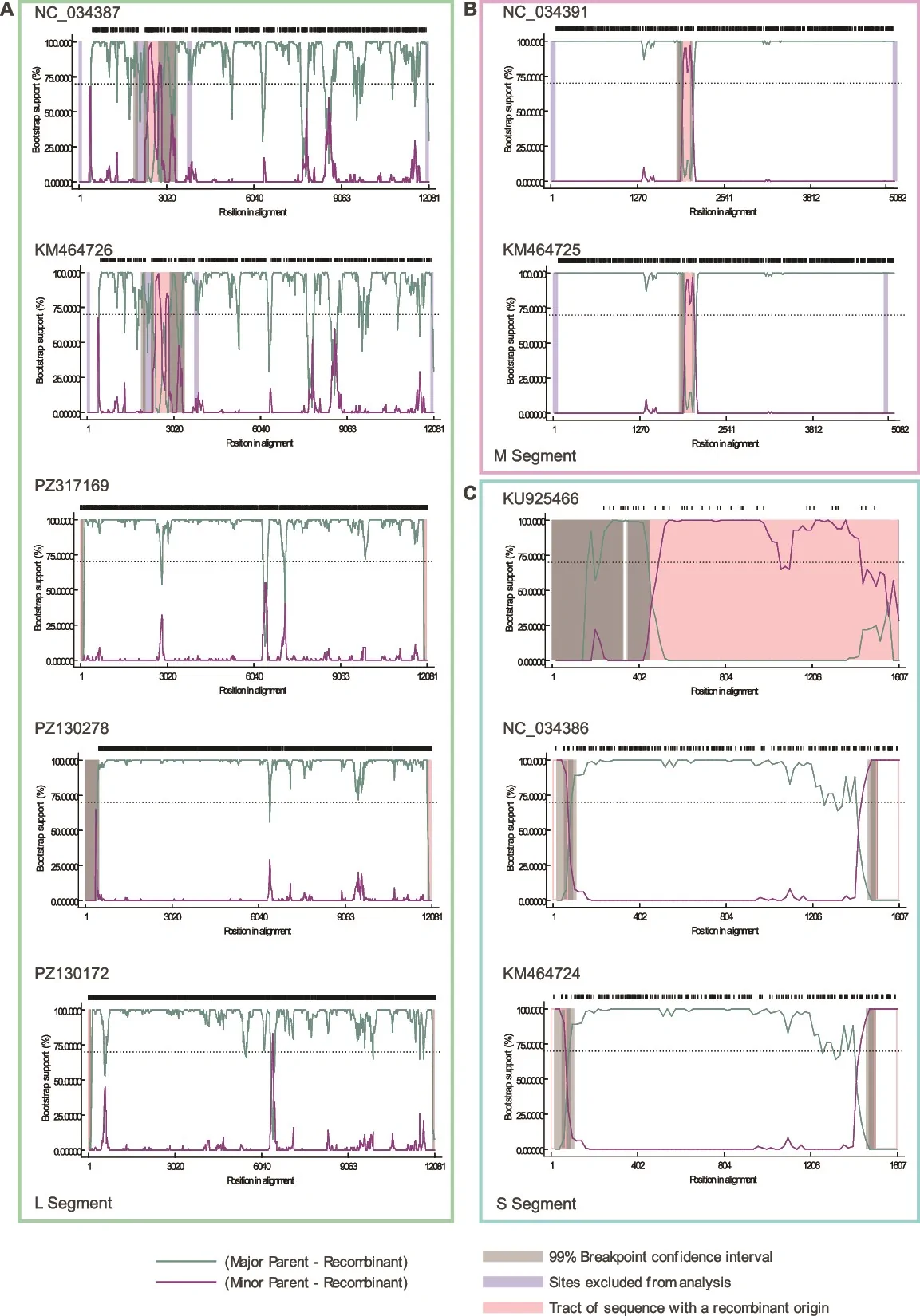
Homologous recombination analysis of NSDV genomic segments. Recombination detection and breakpoint mapping of NSDV L (left panels) and S/M (right panels) genomic segments. Recombinant analysis showing the bootstrap support for recombination (y-axis, 0%–100%) along the aligned nucleotide positions (x-axis) of representative recombinant strains. Coloured traces indicate the major parent (teal) and minor parent (purple) similarity profiles. The grey shaded vertical bars denote the 99% breakpoint confidence interval, while the light purple vertical bars indicate sites excluded from analysis due to low complexity or missing data. Recombination tracts with strong statistical support are highlighted in pink, confirming independent recombination events in both L and S segments.

All L-segment recombinants originated from China (Jilin, Beijing, Shandong) (Fig. 3A, Supplementary Table 4). Two Jilin recombinants (NC_034387, KM464726) shared the same major parent and the same minor parent from Shandong, indicating intra-provincial gene flow. Two additional recombinants (PZ317169, PZ130172) shared a major parent from Henan (OP558304), a sheep-derived isolate, indicating geographic dispersal of recombinant lineages across provinces and transmission between sheep and tick hosts. No foreign parents were involved in L-segment events. The M and S segments displayed evidence of African-lineage parental sequences in Chinese recombinants. The M segment (n = 2) showed a distinct pattern: both Jilin recombinants shared the same major parent from Shandong (OR495600) and the same minor parent from Kenya (EU697952) (Fig. 3B, Supplementary Table 4). RDP4 and Simplot analyses identified strain EU697952 as the closest sampled relative of the minor parent, indicating that the recombination donor belonged to the Kenyan lineage represented by this isolate, rather than EU697952 itself being the direct donor. This pattern suggests that viral lineages with African ancestry contributed to the genetic diversity of Chinese NSDV populations, likely through historical viral movement followed by local recombination events. The S segment (n = 3) displayed the most diverse geographic combination (Fig. 3C, Supplementary Table 4). Two Jilin recombinants shared a major parent from Hubei (MH791451) and a minor parent from Kenya (KT869018), again showing evidence of African-lineage sequences in Chinese recombinants and transmission between saiga and tick hosts. A third S-segment event was confined to India: an Orissa recombinant (KU925466) had both parents from Karnataka state (HQ286605), representing long-term local recombination without foreign involvement.

Geographically, Jilin Province emerged as a recombination hotspot, hosting the largest number of recombinants (6 out of 10 events: 2 L, 2 M, 2 S), followed by Shandong (2 L) and Beijing (1 L). The Shandong tick strain (PZ130278) served as a minor parent for two L-segment recombinants (NC_034387, KM464726) and also appeared as a recombinant itself (MH791449, with Hubei parent), suggesting that Shandong may act as both a donor and recipient of recombination events.

### Selection pressure analysis

After removing the ten confirmed recombinants, we estimated dN/dS ratios for the L, M, and S coding regions to assess overall selective constraints (Fig. 4A-B). The results revealed markedly heterogeneous evolutionary pressures across the three genomic segments. The L segment (encoding RdRp) exhibited a mean dN/dS of 0.365 (Fig. 4B), indicating that it evolves under strong purifying selection, consistent with its essential, highly conserved function in viral replication. In contrast, both the M segment (encoding the glycoprotein precursor) and the S segment (encoding the nucleoprotein) showed mean dN/dS values substantially greater than 1 (3.020 and 5.360, respectively), suggesting that, at the global level, these segments are not predominantly constrained by purifying selection. Site-specific analyses under Model 2 (positive selection) revealed that 76.0% of M-segment codons (1 155/1519) and 77.4% of S-segment codons (359/464) were assigned to positively selected site classes (ω = 3.88 and 6.96, respectively), with the remainder falling into neutral classes (ω = 1.00). BEB analysis (Supplementary Table 5) further identified 316 M-segment codon sites (20.8%) and 57 S-segment sites (12.3%) as positively selected at the 95% posterior probability threshold, including 56 (3.7%) and 33 (7.1%) sites, respectively, at the 99% threshold. Likelihood ratio tests strongly supported Model 2 over nearly neutral Model 1 for both segments (M: 2ΔlnL = 265.40; S: 2ΔlnL = 109.40; df = 2, *P* < 0.001). These results indicate that the high global dN/dS ratios for the M and S genes reflect widespread positive selection distributed across the protein sequences, rather than being driven by a small number of outlier codons. Despite the high global mean dN/dS values for the M and S segments, codon-by-codon analyses (Shannon entropy, Fig. 4C) confirmed that conserved functional domains within these proteins (e.g. the Gc fusion domain in the M segment, and the central core of the N protein in the S segment) remain under strong purifying selection, characterized by low entropy and minimal amino acid variation. Nucleotide substitution rates mirrored these selection patterns: L: ~ 1.56 × 10^−4^, M: ~ 3.54 × 10^−4^, and S: ~ 6.81 × 10^−4^ substitutions per site per year. The S segment evolves approximately four times faster than the L segment and twice as fast as the M segment. Notably, we observed a partial inverse relationship between evolutionary rate and recombination frequency: the slowest-evolving L segment exhibited the highest number of recombination events (n = 5), while the fastest-evolving S segment had fewer recombination events (n = 3; Fig. 4B, C).

**Figure 4:**
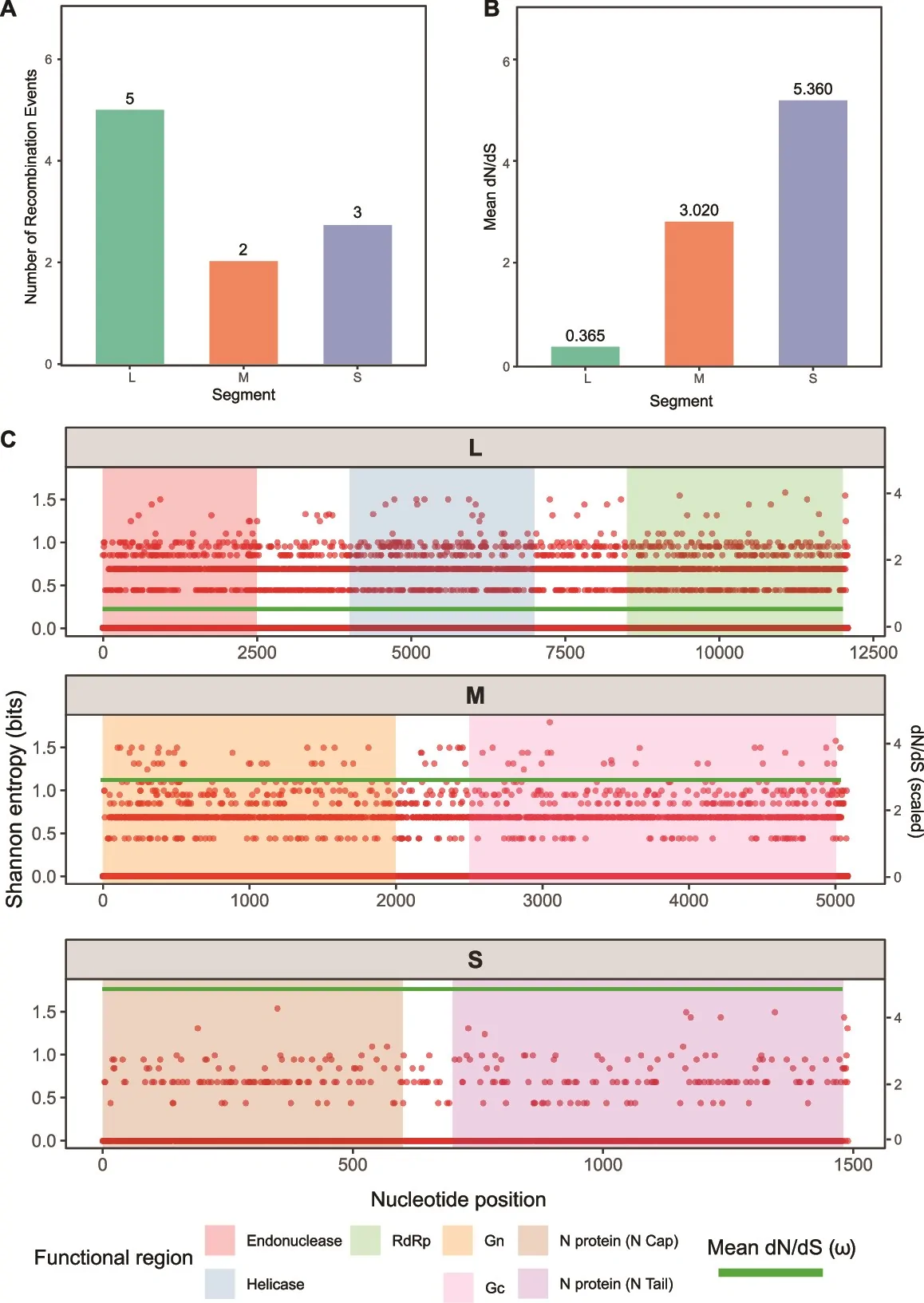
Recombination frequency, selection pressure, and sequence diversity across NSDV genomic segments. Integrated analysis of recombination, selection, and sequence diversity in the three genomic segments (L, M, S) of NSDV. (A) Bar plot showing the total number of confirmed homologous recombination events per segment, with the L segment exhibiting the highest frequency (5 events), followed by M and S segments (2 and 3 events, respectively). (B) Bar plot of mean dN/dS ratios across segments, demonstrating that the L segment shows the strongest purifying selection (lowest dN/dS of 0.365), while the M (3.020) and S (5.360) segments show progressively weaker purifying selection, consistent with relaxed or positive selection pressures. (C) Sliding-window analysis of sequence entropy (red scatter points, left y-axis) and dN/dS ratios (green lines, right y-axis, scaled) across the full length of the L, M, and S segments. Coloured shaded regions at the 5′ end of each segment correspond to key functional domains annotated in the legend. Entropy peaks indicate hypervariable sites, while dN/dS values reflect site-specific selection pressure, with values > 1 indicating positive selection and < 1 indicating purifying selection.

### Temporal and spatial dynamics of the origin and diffusion of NSDV

#### Temporal origin and divergence times

A time-scaled Bayesian phylogenetic analysis of the S segment (the most frequently sequenced segment) estimated the time to the most recent common ancestor (tMRCA) of all NSDV strains to be approximately 1732 Common Era (ce) (95% HPD: 1624–1833, Fig. 5). The mean evolutionary rate was estimated to be 4.07 × 10^−4^ substitutions per site per year (95% HPD: 2.60 × 10^−4^-5.77 × 10^−4^), falling within the range reported for other tick-borne nairoviruses, such as CCHFV (range: 0.34 × 10^−4^ to 4.7 × 10^−4^) (Deyde et al. 2006, Carroll et al. 2010, Zehender et al. 2013). From this ancestral root, the Chinese lineage diverged from the common ancestor of the Indian and Kenyan lineages. The Indian and Kenyan lineages subsequently split from each other around 1763 ce (95% HPD: 1677–1845), with the Kenyan lineage (Lineage C) coalescing more recently (~2004 ce; tMRCA of the two Kenyan samples). The Asia lineage subsequently split into the Indian lineage (divergence ~ 1883 ce, 95% HPD: 1828–1907) and the Chinese lineage (divergence ~ 1934 ce, 95% HPD: 1899–1968). This temporal sequence is consistent with, but does not definitively prove, a stepwise eastward dispersal pattern; however, the root node represents a split between the Chinese lineage and the common ancestor of Indian and Kenyan lineages (not a split between African and Asian lineages). The ancestral location at the root node is unresolved (MAP = India_Karnataka, 0.353; Kenya, 0.302; Supplementary Table 6), and sparse sampling from intermediate regions precludes robust reconstruction of the exact migration path. After its divergence, the Chinese lineage underwent extensive regional radiation from the 1960s onward, generating sub-lineages in Henan, Shandong, Hubei, Liaoning, Heilongjiang, and Beijing (Fig. 5).

**Figure 5:**
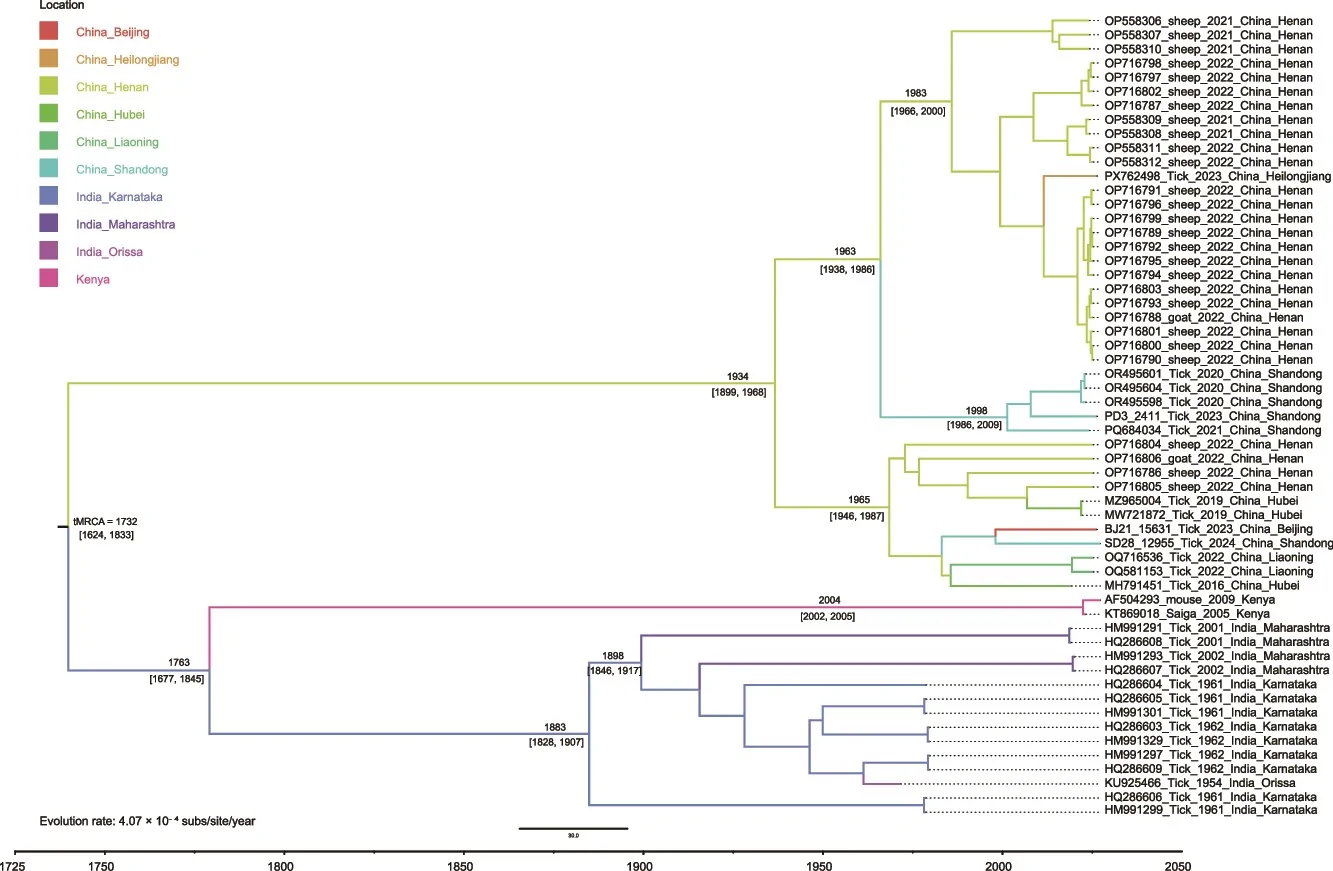
Time-scaled Bayesian phylogeographic analysis of NSDV. Time-calibrated MCC tree inferred from complete NSDV genomic sequences, illustrating the spatiotemporal spread of the virus. Branches are coloured according to the most probable geographic location of each node, as defined in the legend (China: Beijing, Heilongjiang, Henan, Hubei, Liaoning, Shandong; India: Karnataka, Maharashtra, Orissa; Kenya). Node labels indicate the median estimated tMRCA with 95% HPD intervals in brackets. The x-axis represents time in years (1725–2050 ce). The mean evolutionary rate is annotated as 4.07 × 10^−4^ substitutions per site per year.

#### Discrete phylogeographic migration pathways

The BSSVS analysis identified a network of highly supported intra-continental migration routes, while inter-continental routes received limited support (Fig. 6, Supplementary Fig. 5 and Table 7). Notably, intra-continental movements received substantially stronger statistical support and are less susceptible to bias from asymmetric sampling times, representing the most robust findings of our phylogeographic analysis. Within India, a very strong signal was detected from Maharashtra to Karnataka (BF = 114.77, PP = 0.93), indicating intense viral circulation between these two southern states. Within China, the strongest intra-national route was from Hubei to Heilongjiang (BF = 258.05, PP = 0.97), identifying a major dispersal route from central to northeastern China. Hubei also served as an origin for spread to Henan (BF = 24.14, PP = 0.74) and, with lower support, to Beijing, consistent with its role as a central hub. Bidirectional migration between Beijing and Hubei was also detected. Notably, Shandong did not act as a source for other Chinese provinces (all outgoing routes had low support).

**Figure 6:**
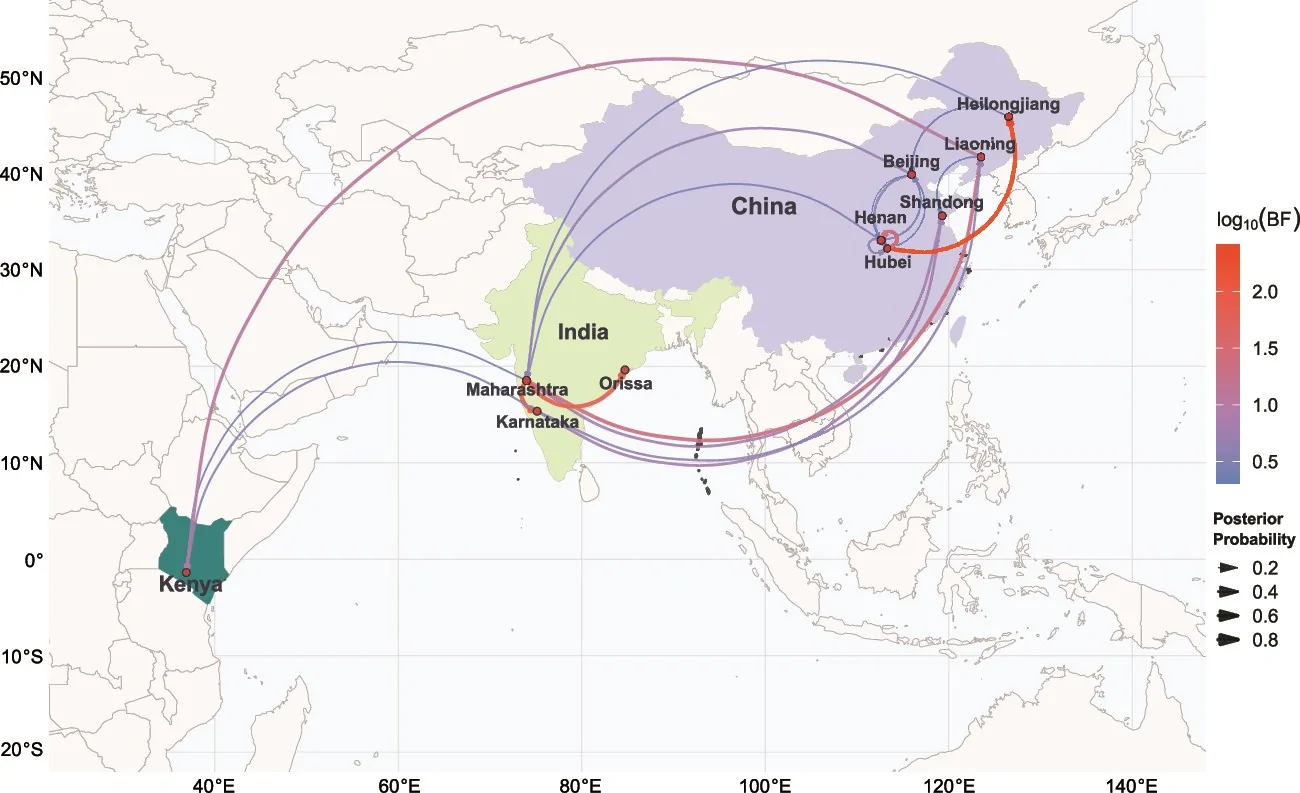
Global spatiotemporal dispersal routes of NSDV. Spatial phylogeographic reconstruction of NSDV transmission routes, inferred from discrete Bayesian phylogeographic analysis. The map shows migration pathways between geographic locations (Kenya; Indian states: Maharashtra, Karnataka, Orissa; Chinese provinces: Henan, Hubei, Shandong, Liaoning, Beijing, Heilongjiang). Only migration routes with BF > 2 (i.e. log_10_(BF) > 0.3), corresponding to ‘substantial’ support, are displayed. Lines represent inferred viral dispersal routes, with line intensity corresponding to the strength of statistical support (log10-transformed Bayes Factor, BF, ranging from 0.3 to 2.0). Arrowhead size of route arrows corresponds to posterior probability (PP), with larger arrows denoting stronger support (PP = 0.2, 0.4, 0.6, 0.8). Circles mark the geographic positions of each sampling location site.

Inter-continental inference was limited by sparse and geographically biased sampling, particularly the small number of available sequences from Kenya. Several putative migration routes from southern India (Maharashtra, Karnataka) to eastern China (Liaoning, Shandong) received moderate support: Maharashtra to Liaoning (BF = 22.57, PP =0.73), Maharashtra to Shandong (BF = 6.40, PP = 0.44), Karnataka to Shandong (BF = 6.50, PP = 0.44), and Karnataka to Liaoning (BF = 4.25, PP = 0.34). Although the estimated timing of these inferred dispersal events (post-1883 ce) aligns with the inferred origin of the Indian lineage, the low posterior probabilities for most routes (PP < 0.5), combined with a strong correlation between sampling location and collection date, undermine confidence in resolving fine-scale viral movement pathways. No direct Kenya–China or Kenya–India migration route attained meaningful statistical support (all BF < 2). A route from Liaoning (China) to Kenya was detected (BF = 11.84, PP = 0.59), but this inference is likely confounded by severe undersampling in Kenyan and the potential existence of unsampled intermediary populations along the transmission chain. These findings are compatible with a stepwise eastward dispersal following the establishment of the Indian lineage (~1883 ce) and preceding the regional radiation of the Chinese lineage (1960s), however, robust inference of trans-continental transmission routes cannot be drawn from the current data.

### Demographic history reconstruction

Bayesian skyline plot analysis revealed three distinct phases (Supplementary Fig. 6). Phase 1 (1830s–1950s) was featured by a prolonged period of moderate but gradually declining effective population size (N_e_), decreasing from ~ 215 to ~ 141. Phase 2 (1958–1962) was featured by a dramatic population collapse, with N_e_ dropping by nearly 40% within 3–4 years. Phase 3 (1960s–2020s) was featured by sustained exponential growth, with N_e_ rebounding to ~ 225 by the mid-1980s, peaking at ~ 244 in the mid-1990s, stabilizing at ~ 240–245 through the 2010s, followed by a modest decline after 2020 (mean N_e_ ~ 78 by the end of 2023).

## Discussion

Although NSDV infections have been widely documented in Asia and Africa, the evolutionary mechanisms underlying their emergence and transmission remain poorly understood. By systematically integrating publicly available genome sequences spanning from the 1950s to 2024, our study provides a comprehensive phylogenetic and phylogeographic analysis of NSDV, revealing three geographically structured lineages (India, China, Kenya) with geographic isolation, rather than host adaptation, as the primary driver of diversification.

Our finding that NSDV strains from different hosts are intermingled within each lineage, with no host-specific clustering, is consistent with previous reports that NSDV strains from ticks and mammals in China are phylogenetically closely related (Yang et al. 2019, Wang et al. 2024). This pattern indicates that frequent cross-species transmission between ticks and vertebrates is a key mechanism maintaining viral genetic diversity (Li et al. 2023), and that host-specific evolutionary constraints are minimal. The Chinese lineage, comprising isolates from multiple provinces (Henan, Hubei, Shandong, Liaoning, Jilin, Beijing), shows further subdivision into at least two subclades, suggesting local diversification within China. Notably, all Chinese NSDV strains identified in this study showed the closest genetic relationship to those previously detected in *H. longicornis* ticks (Gong et al. 2015, Yang et al. 2019), reinforcing the role of *H. longicornis* as the primary vector for NSDV in East Asia (Gong et al. 2015).

Consistent with previous reports that Chinese NSDV strains form a distinct cluster separate from African and Indian strains (Wang et al. 2024). Our expanded dataset (including a larger number of Chinese isolates from Henan, Hubei, Shandong, Liaoning, Jilin, Beijing) confirms this observation and further reveals that at least two sub-clades within the Chinese lineage, suggesting local diversification. The close genetic relationship of these strains to those previously detected in *H. longicornis* ticks in Henan, Hubei, Shandong, and Jilin provinces (Gong et al. 2015, Yang et al. 2019, Wang et al. 2024, Yao et al. 2025) further supports the role of *H. longicornis* as the primary vector for NSDV in East Asia.

Despite notable topological discordance among the L, M, and S segment trees, a pattern often suggestive of reassortment in segmented RNA viruses, formal permutation testing detected no statistically significant inter-segment reassortment among the 18 NSDV isolates analysed. This absence likely reflects the limited genetic diversity of the analysed isolates (reducing the likelihood of co-infection with divergent strains) and strong functional constraints on segment compatibility (Yadav et al. 2011, Zhou et al. 2023, Wang et al. 2024). The four candidate strains identified using a uniform global threshold exhibited elevated cophenetic distance differences but failed to satisfy strain-specific significance criteria (all *P* > 0.05; Supplementary Table 3); they are therefore best interpreted as cases of segment-specific phylogenetic discordance attributable to alternative evolutionary mechanisms, including localized positive selection or incomplete lineage sorting, rather than authentic reassortment events. Crucially, the permutation test confirmed that the observed levels of topological discordance (quantified by RF distances and tanglegram-based incongruence) fall within the null distribution expected under a non-reassortment model, further reinforcing the absence of statistical support for reassortment. Therefore, we conclude that no statistically supported inter-segment reassortment event was detected among the analysed NSDV isolates.

In contrast, homologous recombination appears to be is a major driver of NSDV genetic diversity (Bardossy et al. 2024), with 10 confirmed events identified across the three segments. The L segment exhibited the highest recombination frequency (5 events), consistent with the observation that longer genomic sequences may be more prone to template switching during viral replication (Martin et al. 2015). All identified recombinants were tick-derived, with parental strains originating from ticks, sheep, or saiga antelope. This pattern indicates that recombinant NSDV can be transmitted across host species following the recombination event, rather than that recombination itself occurs across host boundaries. Geographically, Jilin Province hosted the majority of recombination events (6 out of 10: 2 L, 2 M, 2 S), followed by Shandong (2 L) and Beijing (1 L). However, this pattern likely reflects the higher sampling density in Jilin relative to other provinces. The Shandong tick strain (PZ130278) served as a minor parent for two L-segment recombinants (NC_034387, KM464726) and also appeared as a recombinant itself (MH791449, with Hubei parent), suggesting that Shandong may act as both a donor and recipient of recombination events. The M and S segments displayed evidence of African parental sequences in Chinese recombinants, consistent with historical intercontinental viral movement. The M segment (n = 2) showed a distinct pattern: both Jilin recombinants shared the same major parent from Shandong (OR495600) and the same minor parent from Kenya (EU697952) (Fig. 3B, Supplementary Table 4). For both M-segment events, the Kenyan strain EU697952 was identified as the minor parent by RDP4 and Simplot analyses; while this indicates that the recombination donor belonged to the Kenyan lineage represented by this isolate, it does not establish that EU697952 itself was the direct donor strain. The S segment (n = 3) displayed the most diverse geographic combination (Fig. 3C, Supplementary Table 4). Two Jilin recombinants shared a major parent from Hubei (MH791451) and a minor parent from Kenya (KT869018), again showing evidence of African-lineage sequences in Chinese recombinants and transmission between saiga and tick hosts. A third S-segment event was confined to India: an Orissa recombinant (KU925466) had both parents from Karnataka state (HQ286605), representing long-term local recombination without foreign involvement. Notably, the predominance of intra-segment recombination over inter-segment reassortment in NSDV may enable fine-tuning of core viral functional domains without disrupting essential inter-segment compatibility (Simon-Loriere and Holmes 2011), an evolutionary strategy particularly adaptive for a tick-borne virus that must replicate efficiently in both arthropod vectors and vertebrate hosts.

Selection pressure analyses revealed marked differences among the three genomic segments, a pattern commonly observed in segmented RNA viruses, where surface-exposed glycoproteins and immunomodulatory proteins experience stronger immune pressure than conserved internal replicases (Chen 2012). The L segment (encoding the RdRp) exhibited the strongest purifying selection (mean dN/dS = 0.365), consistent with its essential, highly conserved function in RNA replication. Recombination breakpoints in the L segment were largely restricted to the 5′ and 3′ non-coding regions, explaining how this segment can undergo frequent recombination without a corresponding increase in evolutionary rate. The M segment, encoding the glycoprotein precursor, showed an elevated mean dN/dS (3.020). Together with the detection of recombinants in this segment, this finding suggests that positive selection drives amino acid changes in the Gn and Gc glycoproteins, which are critical for host cell attachment, membrane fusion, and immune evasion (Iglesias-Rivas et al. 2025). The elevated dN/dS ratio likely reflects episodic diversifying selection acting on surface-accessible domains exposed to host humoral immunity.

The S segment, encoding the nucleoprotein, exhibited the highest mean dN/dS (5.360) and evolutionary rate (~6.81 × 10^−4^ substitutions/site/year). Among nairoviruses, the nucleoprotein is a major antigenic target, and sequence diversification in the S segment has been linked to immune evasion and cross-reactive antibody responses (Hasing and Pang 2021). This rapid evolution may be driven by positive selection acting on the N protein, which modulates the host interferon response, thereby facing dual pressures from both the adaptive and innate immune systems. We observed a partial inverse relationship between evolutionary rate and recombination frequency: the slowest-evolving L segment experienced the most recombination events, while the fastest-evolving S segment had fewer (n = 3). This pattern may reflect an evolutionary trade-off (Iglesias-Rivas et al. 2025), allowing the virus to maintain genetic stability in essential replication machinery while generating adaptive diversity in immune-related genes (Chen 2012). Notably, the restriction of recombination breakpoints to non-coding regions in the L segment suggests that recombination in this segment is selectively neutral or subject to strong purifying selection, whereas recombination in the M and S segments may occasionally generate novel antigenic variants.

These evolutionary findings complement the prioritized research agenda recently outlined by (Zhang et al. 2026). Positive selection in the M- and S-segment surface proteins and frequent recombination in the L-segment RdRp highlight moving evolutionary targets that inform Tier 1 priorities (receptor/OTU identification), while conserved domains under purifying selection represent promising targets for the broad-spectrum vaccines and antibody therapies proposed in their Tier 3 translational goals. These will be directions for future research.

Our phylogeographic analysis suggests that the geographic origin of NSDV remains unresolved. The temporal sequence of divergence, global tMRCA ~ 1732 ce (initial split between the Chinese lineage and the common ancestor of Indian and Kenyan lineages), followed by internal diversification of the Indian + Kenyan (~1763 ce), Indian (~1883 ce) and Chinese (~1934 ce) lineages, is consistent with stepwise eastward dispersal from an ancestral source. However, no direct phylogeographic migration route from Africa to India received meaningful support (all BF < 3), likely due to missing samples from intermediate regions. South Asia appears to have served as a critical diversification hub for subsequent eastward spread, with moderate phylogeographic support for India-to-China routes (BF = 6.40–22.57) identifying the Indian subcontinent as the primary and immediate source of NSDV introductions into East Asia. Although the root node of NSDV dates to the mid-18th century (~1732 ce), the ancestral geographic location is unresolved; the genetic and geographic diversity of the virus was profoundly shaped in South Asia. The Indian lineage diverged in the late 19th century, approximately 50 years before the diversification of Chinese lineages, positioning the Indian subcontinent as a key source population. This temporal pattern aligns with historical livestock trade routes during the colonial era, which may have facilitated the translocation of infected ticks or livestock from East Africa to India (Krasteva et al. 2020). Notably, direct Kenya-China transmission routes received negligible support, whereas strong signals from India to China identify South Asia as the primary and immediate source of NSDV introductions into East Asia. These well-supported, multiple independent introductions underscore the role of anthropogenic factors (e.g. trade, livestock movement, or migratory birds) in bridging these regions (Linthicum et al. 2016). Within China, Hubei province emerged as a critical geographic hub, acting as the source for the two strongest dispersal corridors: the northward pathway to Heilongjiang and the route to Henan. The exceptional support for the Hubei to Heilongjiang link (BF = 258.05) suggests the presence of an ecological or anthropogenic ‘superhighway’, potentially driven by major transportation networks, livestock distribution channels, or continuous tick habitats. A dispersal route from Liaoning, China to Kenya (BF = 11.84) was detected, but given the very limited Kenyan sampling and the lack of intermediate samples, this inference is tentative and likely confounded by sampling bias. Alternative explanations, including unsampled source populations or indirect transmission *via* intermediate regions, cannot be excluded. Nevertheless, if this signal reflects genuine viral movement, several mechanisms could underlie long-distance dispersal. Over the past two decades, growing international trade in livestock and animal products has created opportunities for tick-borne virus introduction *via* infected animals or associated tick vectors (World Organisation for Animal 2010, Krasteva et al. 2020, Yao et al. 2025). Additionally, increasing Chinese veterinary cooperation and livestock development projects in Kenya and other African nations may facilitate the movement of biological materials and potentially infected vectors. The geographic concentration of this signal in Liaoning—a major livestock production and trade hub in northeastern China—is consistent with a trade-associated dispersal pathway. Migratory birds along Afro-Eurasian flyways represent another potential long-distance dispersal mechanism, though their role in NSDV ecology remains uncharacterized. The mechanisms underlying this dispersal signal, remain unclear and warrant further integration with ecological and traceability data (Krasteva et al. 2020).

Several limitations should be acknowledged. First, the limited number of Kenyan samples may bias inferences of African origins. Second, detected recombination events were not experimentally validated for phenotypic changes. Third, phylogeographic analyses are constrained by sampling locations; the absence of samples from intermediate regions (e.g. Central or Southeast Asia) may bias inferences of transmission pathways and underestimate the role of these areas as potential transmission bridges. Fourth, the mechanisms underlying the China–Kenya transmission route remain unclear and require further ecological investigation. Finally, The Bayesian skyline plot shows a decline after 2020, which might be an artefact of sparse sampling in recent years.

In summary, this study reveals that geographic isolation, rather than host adaptation, drives NSDV lineage diversification. Recombination was detected, but no reassortment was statistically supported among the 18 genomes (limited by sample size). The divergence timeline is consistent with stepwise dispersal, yet the exact origin and a direct Africa–India route lack robust support owing to sparse intermediate sampling and only two Kenyan sequences. These findings provide a basis for refining surveillance and control strategies, including prioritizing high-risk transmission hubs for enhanced tick surveillance, and informing risk assessments for potential introduction into currently unaffected regions.

## Supplementary Material

Supplementary_materials_veag061

## Data Availability

All relevant data are presented in the main figures and supplementary materials. All the raw data included in the current study are publicly available in the GenBank (https://www.ncbi.nlm.nih.gov/). Custom R scripts used for statistical analyses and figure generation are available from the corresponding author upon reasonable request.
