# Supplementary material for: Phylogeography and evolutionary dynamics of Nairobi sheep disease virus
Source: Virus Evol. 2026 Sep 21;12(1):veag061. doi: 10.1093/ve/veag061 (PMC13628517; doi:10.1093/ve/veag061)
Supplement: Supplementary file 1 [file supplementary_materials_veag061.zip › Supplementary_materials_veag061.docx]

# Supplemental Figures

**Supplementary Figure 1. Assessment of temporal signal in the NSDV S-segment dataset using TempEST.**

Scatter plot of root-to-tip divergence against sampling time (1954–2024). The linear regression shows a strong clock-like signal (correlation coefficient r = 0.94, *R*² = 0.89, residual mean square ≈ 7.76×10⁻⁴), supporting the use of a strict molecular clock model.

**Supplementary****Figure 2. Phylogenetic analysis of the L segment of Nairobi sheep disease virus (NSDV).**

Maximum-likelihood phylogenetic tree of complete L segment sequences of NSDV, constructed using the GTR+G+I nucleotide substitution model with 1,000 bootstrap replicates. The tree is rooted at the midpoint, and bootstrap values ≥70% are shown at the nodes. The scale bar represents 0.1 nucleotide substitutions per site. Three distinct phylogenetic lineages are annotated: Lineage A (India lineage, purple), Lineage C (Kenya lineage, green), and Lineage B (China lineage, red). Sequence labels include GenBank accession number, host/sample type, isolate name, collection year, and geographic origin.

**Supplementary Figure 3. Phylogenetic analysis of the M segment of Nairobi sheep disease virus (NSDV).**

Maximum-likelihood phylogenetic tree of complete M segment sequences of NSDV, constructed using the GTR+G+I nucleotide substitution model with 1,000 bootstrap replicates. The tree is rooted at the midpoint, and bootstrap values ≥70% are shown at the nodes. The scale bar represents 0.1 nucleotide substitutions per site. Three distinct phylogenetic lineages are annotated: Lineage A (India lineage, purple), Lineage C (Kenya lineage, green), and Lineage B (China lineage, red). Sequence labels include GenBank accession number, host/sample type, isolate name, collection year, and geographic origin.

**Supplementary Figure 4. Phylogenetic analysis of the S segment of Nairobi sheep disease virus (NSDV).**

Maximum-likelihood phylogenetic tree of complete S segment sequences of NSDV, constructed using the GTR+G+I nucleotide substitution model with 1,000 bootstrap replicates. The tree is rooted at the midpoint, and bootstrap values ≥70% are shown at the nodes. The scale bar represents 0.1 nucleotide substitutions per site. Three distinct phylogenetic lineages are annotated: Lineage A (India lineage, purple), Lineage C (Kenya lineage, green), and Lineage B (China lineage, red). Sequence labels include GenBank accession number, host/sample type, isolate name, collection year, and geographic origin.

**Supplementary Figure 5. Global spatiotemporal dispersal routes of Nairobi sheep disease virus (NSDV).**

Spatial phylogeographic reconstruction of NSDV transmission routes, inferred from discrete Bayesian phylogeographic analysis. The map illustrates statistically supported migration pathways between geographic locations (Kenya; Indian states: Maharashtra, Karnataka, Orissa; Chinese provinces: Henan, Hubei, Shandong, Liaoning, Beijing, Heilongjiang). Lines represent inferred viral dispersal routes, with color intensity corresponding to the strength of statistical support (log₁₀-transformed Bayes Factor, BF), ranging from 0.5 (blue, low support) to 2.0 (red, high support), as indicated by the color bar. The size of route arrows corresponds to posterior probability (PP), with larger arrows denoting stronger support (PP = 0.2, 0.4, 0.6, 0.8). Red circles mark the geographic positions of each sampling location.

**Supplementary Figure 6. Bayesian skyline plot of NSDV effective population size through time.**

Bayesian skyline plot (BSP) illustrating the temporal dynamics of the effective population size (N_e_) of Nairobi sheep disease virus (NSDV) from the 18th century to the present. The solid blue line represents the median estimated N_e_, and the light blue shaded area denotes the 95% highest posterior density (HPD) credible interval. The y-axis is plotted on a logarithmic scale, showing N_e_ values ranging from 10 to 1000, while the x-axis represents time in calendar years.
